# German Aortic Valve Score in Risk Assessment for Surgical Aortic Valve Replacement in a Brazilian Center

**DOI:** 10.21470/1678-9741-2019-0373

**Published:** 2020

**Authors:** Sérgio C. Rayol, Michel Pompeu B. O. Sá, Luiz Rafael P. Cavalcanti, Roberto G. S. Diniz, Álvaro M. Perazzo, Antônio C. A. Escorel Neto, Konstantin Zhigalov, Arjang Ruhparwar, Alexander Weymann, Ricardo C. Lima

**Affiliations:** 1 Division of Cardiovascular Surgery of Pronto-Socorro Cardiológico de Pernambuco - PROCAPE, Recife, Brazil.; 2 University of Pernambuco - UPE, Recife, Brazil.; 3 Nucleus of Postgraduate and Research in Health Sciences of Faculty of Medical Sciences and Biological Sciences Institute - FCM/ICB, Recife, Brazil.; 4 Department of Thoracic and Cardiovascular Surgery, West German Heart and Vascular Center Essen, University Hospital of Essen, University Duisburg-Essen, Essen, Germany.

**Keywords:** Aortic Valve, Transcatheter Aortic, Area Under Curve, Confidence Intervals, Calibration, ROC Curve, Sensitivity and Specificity, Heart Valve Prosthesis

## Abstract

**Objective:**

To test the German Aortic Valve (GAV) score at our university hospital in patients undergoing isolated aortic valve replacement (AVR).

**Methods:**

A total of 224 patients who underwent isolated conventional AVR between January 2015 and December 2018 were included. Patients with concomitant procedures and transcatheter aortic valve implantation were excluded. Patients’ data were collected and analyzed retrospectively. Patients’ risk scores were calculated according to criteria described by GAV score. Sensitivity, specificity, and accuracy (area under the ROC curve [AUC]) were also calculated. The calibration of the model was tested by the Hosmer-Lemeshow method.

**Results:**

The mortality rate was 8.04% (18 patients). The patients’ mean age was 58.2±19.3 years and 25% of them were female (56 patients). Mean GAV score was 1.73±5.86 (min: 0.0; max: 3.53). The GAV score showed excellent discriminative capacity (AUC 0.925, 95% confidence interval 0.882-0.956; *P*<0.001). The cutoff “1.8” turned out to be the best discriminatory point with the best combination of sensitivity (88.9%) and specificity (75.7%) to predict operative death. Hosmer-Lemeshow method revealed a *P*-value of 0.687, confirming a good calibration of the model.

**Conclusion:**

The GAV score applies to our population with high predictive accuracy.

**Table:** 

Abbreviations, acronyms & symbols				
AUC	= Area under the ROC curve		LR	= Likelihood ratio
AVR	= Aortic valve replacement		LVEF	= Left ventricular ejection fraction
CI	= Confidence interval		NYHA	= New York Heart Association
COPD	= Chronic obstructive pulmonary disease		ROC	= Receiver operating characteristic
EuroSCORE	= European System for Cardiac Operative Risk Evaluation		SPSS	= Statistical Package for the Social Sciences
GAV	= German Aortic Valve		TAVI	= Transcatheter aortic valve implantation

## INTRODUCTION

The assessment of operative risk is mandatory for all cardiac procedures, since patients need to be informed preoperatively about the risks and surgeons must weigh up pros and cons of a certain procedure. In this scenario, risk scoring systems are used to predict and evaluate results.

Although there are widely spread risk scores, such as the European System for Cardiac Operative Risk Evaluation (EuroSCORE)^[1]^, that have demonstrated good predictive accuracy in the field of cardiovascular surgery, the trend of the moment is for more specific scores to be applied to more specific contexts in cardiac surgery.

Könning et al.^[2]^ published in 2013 the German Aortic Valve (GAV) score. It was designed for fair and reliable outcome evaluation, allows comparison of predicted and observed mortality for conventional aortic valve replacement (AVR) and transcatheter aortic valve implantation (TAVI) in low-, moderate-, and high-risk groups, enables a risk-adjusted benchmark of outcome, and fosters the efforts for continuous improvement of quality in aortic valve procedures.

Since the score has never been tested in Brazil, we aimed to validate the GAV score in patients who underwent conventional AVR at a Brazilian center.

## METHODS

**Fig. 1:**
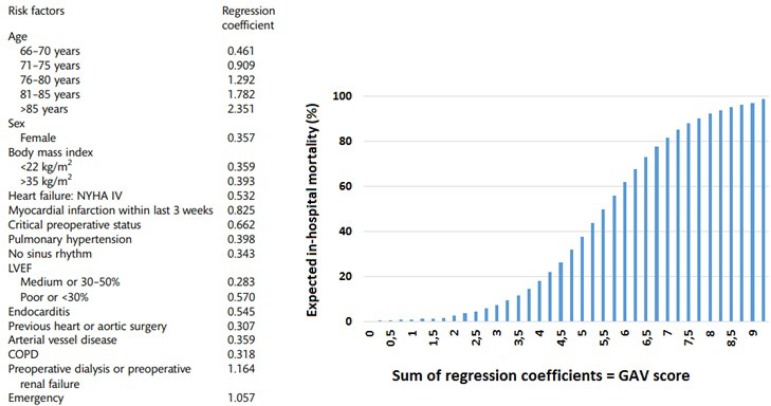
German Aortic Valve (GAV) score. COPD=chronic obstructive pulmonary disease; LVEF=left ventricular ejection fraction; NYHA=New York Heart Association

Patients who underwent conventional AVR between January 2015 and December 2018 were included in the study. Those who underwent concomitant procedures or TAVI were excluded. Data were collected and analyzed retrospectively. Primary endpoint was in-hospital mortality. Patients’ GAV scores were calculated according to the criteria described by Kötting et al.^[2]^ (Figure 1). The score is calculated through the sum of regression coefficients, which corresponds to a certain expected operative mortality.

Sensitivity and specificity were assessed through the receiver operating characteristic (ROC) curve. The discrimination measures the capacity of a model (in this case, the GAV score) to differentiate between the individuals of a sample who suffer an event (in this case, death) from those who do not. The discriminative capacity of the model was estimated by means of the area under the ROC curve (AUC). Calibration of the GAV score was assessed by the Hosmer-Lemeshow test. The calibration is considered to be poor if the test is statistically significant. For the analysis, the Statistical Package for the Social Sciences (SPSS)^®^ software (SPSS, Inc., Chicago, IL, United States of America), version 15.0, for Windows^®^ was used. *P*-values < 0.05 were considered statistically significant.

## RESULTS

We evaluated 224 isolated AVR procedures in adult patients. The mortality rate was 8.04% (18 patients). The patients’ mean age was 58.2±19.3 years and 25% of them were female (56 patients).

**Fig. 2:**
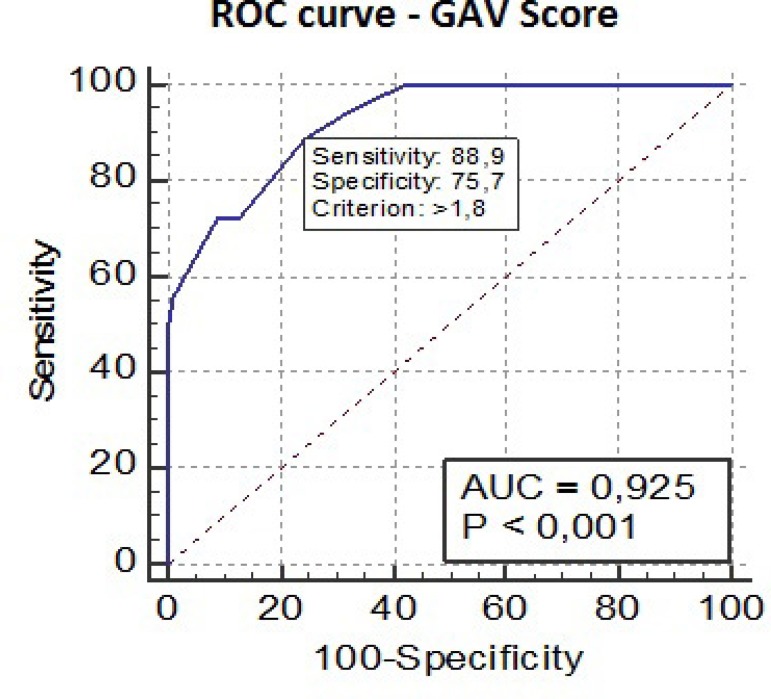
Receiver operating characteristic (ROC) curve - Accuracy. AUC=area under the ROC curve; GAV=German Aortic Valve

Mean GAV score was 1.73±5.86 (min: 0.0; max: 3.53). The GAV score showed excellent discriminative capacity (AUC 0.925, 95% confidence interval [CI] 0.882-0.956; *P*<0.001) (Figure 2). The calibration of the model was tested by the Hosmer-Lemeshow method. The derived *P*-value of 0.687 confirmed a valid accordance of predicted and observed mortality, which means good calibration of the model.

The cutoff “1.8”turned out to be the best discriminatory point with the best combination of sensitivity (88.9%) and specificity (75.7%) to predict operative death (Table 1).

**Table 1:** Best discriminatory point of the German Aortic Valve score.

Score	Sensitivity	95% CI	Specificity	95% CI	+LR	-LR
≥ 0.4	100	81.5 - 100	-	0.0 - 1.8	1	
> 1.1	100	81.5 - 100	57.77	50.7 - 64.6	2.37	-
> 1.4	94.44	72.7 - 99.9	67.96	61.1 - 74.3	2.95	0.082
> 1.8	88.89	65.3 - 98.6	75.73	69.3 - 81.4	3.66	0.15
> 2.3	72.22	46.5 - 90.3	87.38	82.1 - 91.6	5.72	0.32
> 2.9	72.22	46.5 - 90.3	91.26	86.5 - 94.7	8.27	0.30
> 3.7	55.56	30.8 - 78.5	99.03	96.5 - 99.9	57.22	0.45
> 4.7	50	26 - 74	100	98.2 - 100		0.50
> 11.9	-	0.0 - 18.5	100	98.2 - 100		1

CI=confidence interval; ± LR=positive/negative likelihood ratio

## DISCUSSION

The presumption that a scoring system might be comprehensive enough for all patients and cardiovascular surgical procedures could not be further from the truth^[3,4]^. For instance, the widely used EuroSCORE was based on a data set consisting mainly of coronary artery bypass surgeries. Thus, such score might be less well adapted to aortic procedures than a specific score as the one evaluated in the present study. Such aspects have been highlighted by other authors as well^[5-8]^.

Kalender et al.^[9]^ tested for the first time the GAV score out of Germany, studying only 35 isolated AVR procedures in adult patients in Turkey. The patients’ mean age was 61.14±13.25 years (range 29-80 years). The number of female patients was 14 (40%). Mean GAV score was 1.05±0.96 (min: 0; max: 4.98) and mean EuroSCORE II was 2.30±2.60 (min: 0.62, max: 2.30). The GAV score scale showed modest discriminative capacity (AUC 0.647, 95% CI 0.439-0.854).

To the best of our knowledge, our study is the first one to report the results of the GAV score in a Latin American scenario. It is well known that predictive models work best in the series at the location where it was developed. For this reason, the GAV score fits best to the population in Germany. Nevertheless, despite the differences between German and Brazilian populations, the score also showed a very good discriminative capacity in our population.

### Limitation

The major limitations of our study were its non-randomized and retrospective design, single institution setting, and the fact that our hospital is a multi-surgeon one.

## CONCLUSION

The GAV score applies to our population with high predictive accuracy and could be used in our population to calculate operative risk.

**Table:** 

Authors' roles & responsibilities	
SCR	Substantial contributions to the conception or design of the work; or the acquisition, analysis, or interpretation of data for the work; drafting the work or revising it critically for important intellectual content; agreement to be accountable for all aspects of the work in ensuring that questions related to the accuracy or integrity of any part of the work are appropriately investigated and resolved; final approval of the version to be published
MPBOS	Substantial contributions to the conception or design of the work; or the acquisition, analysis, or interpretation of data for the work; drafting the work or revising it critically for important intellectual content; agreement to be accountable for all aspects of the work in ensuring that questions related to the accuracy or integrity of any part of the work are appropriately investigated and resolved; final approval of the version to be published
LRPC	Substantial contributions to the conception or design of the work; or the acquisition, analysis, or interpretation of data for the work; drafting the work or revising it critically for important intellectual content; final approval of the version to be published
AMP	Substantial contributions to the conception or design of the work; or the acquisition, analysis, or interpretation of data for the work; final approval of the version to be published
RGSD	Substantial contributions to the conception or design of the work; or the acquisition, analysis, or interpretation of data for the work; final approval of the version to be published
ACEAN	Substantial contributions to the conception or design of the work; or the acquisition, analysis, or interpretation of data for the work; final approval of the version to be published
KZ	Substantial contributions to the conception or design of the work; or the acquisition, analysis, or interpretation of data for the work; final approval of the version to be published
AR	Substantial contributions to the conception or design of the work; or the acquisition, analysis, or interpretation of data for the work; final approval of the version to be published
AW	Substantial contributions to the conception or design of the work; or the acquisition, analysis, or interpretation of data for the work; final approval of the version to be published
RCL	Substantial contributions to the conception or design of the work; or the acquisition, analysis, or interpretation of data for the work; final approval of the version to be published
